# Pharmacokinetics, Pharmacodynamics and Drug–Drug Interactions of New Anti-Migraine Drugs—Lasmiditan, Gepants, and Calcitonin-Gene-Related Peptide (CGRP) Receptor Monoclonal Antibodies

**DOI:** 10.3390/pharmaceutics12121180

**Published:** 2020-12-03

**Authors:** Danuta Szkutnik-Fiedler

**Affiliations:** Department of Clinical Pharmacy and Biopharmacy, Poznań University of Medical Sciences, Św. Marii Magdaleny 14 St., 61-861 Poznań, Poland; dszkutnik@ump.edu.pl

**Keywords:** migraine, lasmiditan, gepants, monoclonal antibodies, drug–drug interactions

## Abstract

In the last few years, there have been significant advances in migraine management and prevention. Lasmiditan, ubrogepant, rimegepant and monoclonal antibodies (erenumab, fremanezumab, galcanezumab, and eptinezumab) are new drugs that were launched on the US pharmaceutical market; some of them also in Europe. This publication reviews the available worldwide references on the safety of these anti-migraine drugs with a focus on the possible drug–drug (DDI) or drug–food interactions. As is known, bioavailability of a drug and, hence, its pharmacological efficacy depend on its pharmacokinetics and pharmacodynamics, which may be altered by drug interactions. This paper discusses the interactions of gepants and lasmiditan with, i.a., serotonergic drugs, CYP3A4 inhibitors, and inducers or breast cancer resistant protein (BCRP) and P-glycoprotein (P-gp) inhibitors. In the case of monoclonal antibodies, the issue of pharmacodynamic interactions related to the modulation of the immune system functions was addressed. It also focuses on the effect of monoclonal antibodies on expression of class Fc gamma receptors (FcγR).

## 1. Introduction

Migraine is a chronic neurological disorder characterized by a repetitive, usually unilateral, pulsating headache with attacks typically lasting from 4 to 72 h. The pain is characterized by a varying degree of intensity and frequency of occurrence and is accompanied, among others, by photophobia, phonophobia, osmophobia, and nausea and vomiting [1,2]. An average of 11–12% of the population in Europe and North America suffer from migraines, of which 75% are women. Chronic migraine occurs through the chronification of episodic migraine, thus increasing the frequency of attacks and the accompanying change of nature of some of them into pain more reminiscent of a tension-type headache than migraine. Patients with chronic migraine also very often develop drug-overuse headache, which is usually very difficult to differentiate from primary headache [2].

As shown in observational studies in the current epidemiological situation, headache can also be a quite vital symptom in patients with COVID-19, appearing both in presymptomatic and symptomatic phases [2,3,4,5,6]. It was observed that from 11% to 34% of hospitalized patients (mainly young women under 50 years of age) infected with SARS-CoV-2 reported headaches similar to typical migraines or tension headaches. Mean incidence of headaches in all symptomatic COVID-19 patients is approximately 8% [3,4,5,6]. The probable pathophysiology of headache development in COVID-19 patients is associated with neurogenic inflammation in the olfactory and trigeminal nerves due to release of pro-inflammatory mediators, e.g., cytokines and chemokines, as well as activation of prostaglandins in response to penetration of SARS-CoV-2 into the body through the nasal passages [7,8]. 

Current guidelines for treatment of mild to moderate migraine attacks [9,10,11,12] recommend non-opioid analgesics. In moderate to severe attacks, usually triptans alone or in combination therapy with non-steroidal anti-inflammatory drugs (NSAIDs) or paracetamol and antiemetics are recommended. However, due to their vasoconstriction effect, triptans are contraindicated in patients with ischemic heart disease or peripheral vascular disease. Neither do they yield satisfactory results in approximately 30% of patients with severe and moderate migraine [3,13]. Currently, promising novelties in moderate and severe migraine therapy include: lasmiditan (selective 5-HT1F receptor agonist) [13,14], gepants (calcitonin gene-related peptide (CGRP) receptor antagonists) [15], and, in prevention of migraine attacks, anti-CGRP monoclonal antibodies (mAbs) [16,17]. As has been shown so far, all of the above anti-migraine medications can be used in patients with migraine and COVID-19 [3,5,6,8,18]. 

Drug–drug interactions (DDIs) in the pharmacokinetic phase can significantly affect blood concentration and bioavailability of a drug and, thus, its safety and efficacy [19,20]. Pharmacodynamic drug–drug interactions, such as acting as an agonist or antagonist at the receptor, may also increase or decrease the effects of a drug. The risk of interaction increases with each new drug being taken. If two drugs are used simultaneously, there is already a clinically significant risk of interaction; if there are more than seven drugs, interaction is relatively certain [19]. This is of particular importance in the context of the ever-increasing number of chronically ill patients and aging population. After oral administration, the factor determining the occurrence of DDI is mainly drug metabolism mediated by the cytochrome P450 (CYP) system [20] and efflux transporters such as P-glycoprotein (P-gp), the multidrug resistance protein 2 (MRP2), and the breast cancer resistance protein (BCRP) [19]. Significant pharmacokinetic interactions of orally administered drugs may also occur during the absorption phase. In this case, the effect is a decrease rather than an increase in the drug absorption, and a distinction must be made between interactions resulting in a reduced absorption rate and those affecting the total amount of the drug absorbed [19,20]. 

Treatment of migraine, especially of moderate and severe intensity, is often based on polypharmacotherapy and the need to use not only typical analgesics but also such agents as sedatives, hypnotics, or antiemetics [1,2]. Therefore, the risk of DDIs in migraine therapy itself, even without concomitant diseases, may be significantly high. 

This review presents information on safety and possible pharmacokinetic and pharmacodynamics interactions of the newest drugs used to stop migraine attacks, i.e., lasmiditan, ubrogepant, and rimegepant, as well as anti-CGRP monoclonal antibodies administered to prevent migraine.

## 2. Lasmiditan

**Figure 1:**
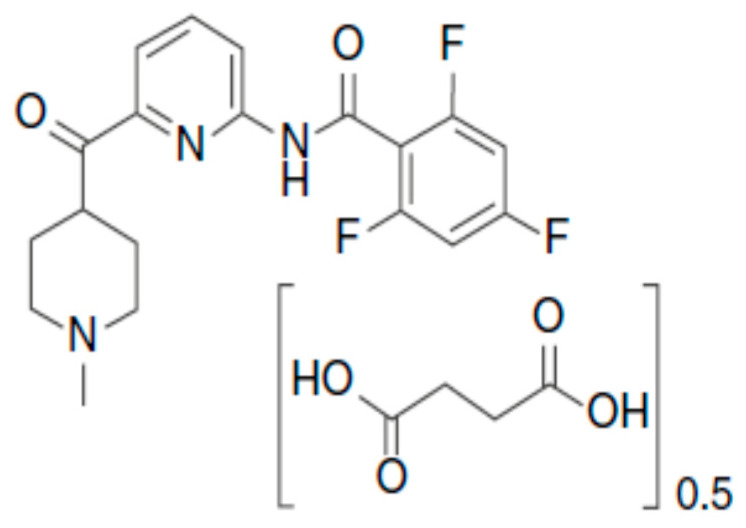
Chemical structure of lasmiditan [14].

For acute treatment of migraine with or without aura in adults, the U.S. Food and Drug Administration (FDA) approved in October 2019 the first-in-class “ditan”—lasmiditan 50 mg and 100 mg tablets [14]. The chemical name of lasmiditan is 2,4,6-trifluoro-N-[6-(1-methylpiperidine-4-carbonyl)pyridin-2-yl]benzamide. The chemical structure is presented in Figure 1 [21]. Lasmiditan is a 5-HT agonist with an over 440 times more potent binding affinity for 5-HT_1F_ than 5-HT_1B_ and 5-HT_1D_ receptors [21] and which potently inhibits markers of electrical stimulation in the trigeminal ganglion [14]. This inhibits release of neuropeptides and neurotransmitters such as CGRP and glutamate, thereby inhibiting their local activity and migraine attack pain pathways [21]. Efficacy and safety of lasmiditan in stopping migraine attacks were confirmed compared to a placebo in randomized phase III clinical trials [22,23,24,25,26,27,28,29,30,31,32,33,34,35,36,37,38,39,40,41,42,43], also in patients with vascular risk factors [27]. Since the 5-HT_1F_ receptor is located mainly in the trigeminal nerve and not in the vascular muscle, such as 5-HT_1B_ and 5-HT_1D_ receptors, lasmiditan does not have a vasoconstriction effect, unlike triptans. Therefore, it is believed that it may be used in patients with cardiovascular diseases [14,21,24,27,29,37,42].

### 2.1. Pharmacokinetics and Pharmacodynamics

Upon oral administration, lasmiditan is rapidly absorbed, reaching peak plasma concentrations in a median of 1.8 h [14,28,29,30,31]. No difference in absorption and bioavailability of lasmiditan was demonstrated during a migraine attack and during the interictal period. Taking the drug with a high-fat meal may prolong the median time to reach maximum plasma concentration (T_max_) by about one hour and increase its exposure (maximum plasma concentration (C_max_) and surface area under the concentration–time curve (AUC) by 22% and 19%, respectively). According to the prescribing information of lasmiditan, however, these differences in exposure are not expected to be clinically significant [28]. 

The binding of lasmiditan to blood proteins is 55%–60%, and biological half-life (T_0.5_) is 5.7 h [14,28]. No accumulation was observed with daily administration. Lasmiditan is primarily eliminated via metabolism, with the major pathway being ketone reduction. Renal excretion plays a minor role in drug clearance. The metabolism of lasmiditan is carried out mainly by non-cytochrome P450 (CYP) enzymes hepatically and extrahepatically. Major metabolites include M3, M7 (oxidation of piperidine ring), M8, (*S*,*R*)-M18 and (*S*,*S*)-M18 (combination of M7 and M8 pathways). M7 and M18 are considered pharmacologically inactive. The following enzymes are not involved in its metabolism: monoamine oxidases, CYP450 reductase, xanthine oxidase, alcohol dehydrogenase, aldehyde dehydrogenase, and aldo-keto reductases [30,42]. Unchanged lasmiditan in urine is around 3% of the dose. In contrast, most of the drug is excreted as metabolite S-M8 (66% of the dose), with the majority of recovery within 48 h after administration [14,28,30]. It has been shown that pharmacokinetics of lasmiditan is not significantly affected by age, sex, race/ethnicity, and body weight [28,30,31,35]. In geriatric patients (65 years of age and older), however, a clinically insignificant increase in C_max_ and AUC was noted (by 21% and 26%, respectively), compared to patients aged 45 or younger [28,30,32]. 

In patients with severe renal impairment (eGFR < 30 mL/min/1.73 m^2^), AUC and C_max_ of lasmiditan were 18% and 13% higher, respectively. The C_max_ and AUC for the major metabolite M8 were 1.2-fold and 2.5-fold greater, for (S,R)-M18 they were 1.4-fold and 2.6-fold greater, and for M7—1.2-fold and 1.7-fold greater, respectively. The metabolites are considered inactive. Considering the chronic-intermittent nature of lasmiditan dosing, increased metabolite exposure may not be clinically relevant and, thus, no dose adjustment was required based on renal function [14,28,33,42]. In addition, in subjects with mild and moderate hepatic impairment (Child–Pugh Class A and B), changes in exposure of lasmiditan were not clinically significant. Lasmiditan exposure (C_max_ and AUC) was 19% and 11%, and 33% and 35% greater in subjects with mild and moderate hepatic impairment, respectively, compared to subjects with normal hepatic function. In patients with severe hepatic impairment (Child–Pugh Class C), lasmiditan has not been studied and is, therefore, not recommended [14,28,30,34,35,42]. 

The recommended single dose of lasmiditan ranges from 50 mg to 200 mg, depending on pain intensity. As shown for lasmiditan, therapeutic gain for 2 h pain freedom was 15–21% (depending on dose) compared to ubrogepant (8–10%) and rimegepant (7%) [24]. It is not recommended to take more than one dose a day or drive vehicles up to 8 h after taking the drug. The most common side effects are dizziness, fatigue, paresthesia, and sedation [14,28,31,32,36,37,38,39,42,43]. 

### 2.2. Interactions with Serotonergic Drugs 

Since lasmiditan is a direct serotonin receptor agonist when administered concomitantly with other prescription and over-the-counter drugs or herbal supplements that also increase 5-HT levels the risk of serotonin syndrome increases [14,28,31,35,36,37,38]. Among these drugs are those which increase serotonin formation (e.g., 5-hydroxytryptophan), increase release of serotonin (e.g., mirtazapine), impair serotonin reuptake from the synaptic cleft into the presynaptic neuron (e.g., meperidine, tramadol, pentazocine, St. John’s wort, dextromethorphan, selective serotonin reuptake inhibitors, selective serotonin–noradrenaline reuptake inhibitors, tricyclic antidepressants), inhibit serotonin metabolism by inhibition of monoamine oxidase (MAO) (e.g., MAO inhibitors), and are direct serotonin receptor agonists (e.g., triptans, fentanyl), or increase sensitivity of postsynaptic serotonin receptor (e.g., lithium) [44]. In the study of Berg et al. [40], however, coadministration of lasmiditan and sumatriptan did not cause serotonin syndrome or any clinically relevant interaction between these two drugs. 

Yet, it is especially important to remember that patients should be carefully monitored for symptoms of serotonin syndrome when doses of ditan and/or other serotonergic drugs are increased [14,28,44,45,46]. Symptoms of serotonin syndrome include, but are not limited to, mental status changes (e.g., irritability, hallucinations, coma), autonomic dysfunction (e.g., tachycardia, hyperthermia, blood pressure lability), neuromuscular abnormalities (e.g., hyperreflexia, incoordination), and/or gastrointestinal symptoms (e.g., nausea, vomiting, diarrhea). The onset of symptoms usually occurs within minutes to hours of receiving a new or a higher dose of serotonergic agent [44]. 

Due to the fact that some drugs may have an extended T_0.5_ (e.g., vortioxetine 66 h), and patients may have hepatic dysfunction which prolongs T_0.5_ of many drugs (e.g., meperidine from 2–4 to 7–11 h), the risk of serotonin syndrome also exists during their sequential administration [44,45,46]. Therefore, during therapy with lasmiditan and other serotonergic drugs, their T_0.5_ values should always be checked and considered. In addition, one should always pay attention to the fact that the drug’s total elimination period is approximately equal to ten times T_0.5_. If serotonin syndrome develops during treatment, all serotonergic medications should be discontinued immediately, and supportive care should be given as needed. Severe cases should be managed under consultation with a toxicologist and may require sedation, neuromuscular blocking drugs, intubation, and mechanical ventilation [44,46].

### 2.3. Interactions with P-gp and BCRP Substrates

Lasmiditan exhibited in vitro inhibition of intestinal P-gp and BCRP with drug–drug interaction indices I_gut_/IC_50_ (intestinal luminal concentration estimated as dose/250 mL/half-maximal inhibitory concentration) of 25 and 16, respectively [42]. These values exceeded the FDA [47] cutoff value of 10, indicating that lasmiditan has the potential to inhibit P-gp or BCRP in vivo [42]. No clinical DDI studies were conducted to evaluate the clinical significance of these DDIs. Concomitant use of lasmiditan (perpetrator) and drugs that are P-gp substrates (victims) may increase their blood levels and cause side effects [14,29,42]. The mechanism involves enhanced absorption and reduced excretion of P-gp substrates due to inhibition of P-gp efflux transporter in the intestine, renal proximal tubule, and liver [44]. Therefore, administration of lasmiditan with P-gp and BCRP substrate drugs should be avoided [14,28,29,42]. 

In vitro studies with the following membrane transporters: multidrug and toxin extrusion proteins (MATE1/MATE2-K), organic cation transporters (OCT1, OCT2), organic anion transporters (OAT1, OAT3), organic anion transporting polypeptide (OATP1B1, or OATP1B3) demonstrated that lasmiditan had a low potential for interactions [14,42]. In a drug–drug interaction study with OCT1 substrate sumatriptan, no change in sumatriptan pharmacokinetics was noted [40,42].

### 2.4. Interactions with Heart Rate Lowering Drugs

It has been shown that in healthy subjects lasmiditan at a single dose of 200 mg, when coadministered with propranolol, could reduce heart rate by additional five beats per minute for a mean maximum of 19 beats per minute compared to propranolol alone [27,28]. Tsai et al. [27] demonstrated that the heart rate decreased shortly after coadministration of lasmiditan with propranolol and was significantly lower for up to 12 h than upon administration of propranolol alone. Compared with administration of either drug alone, however, this combination was generally well-tolerated. There were no significant differences in frequency, severity, or nature of adverse effects. Neither were there any changes observed in the maximum plasma concentration and other pharmacokinetic parameters of lasmiditan, compared to lasmiditan alone. 

In vitro studies have shown that lasmiditan has no vasoconstrictive effects at concentrations up to 100 µM in the rabbit saphenous ring assay which is a reliable predictor of human coronary artery vasoconstrictor liability [42]. Likewise, in in vivo preclinical studies, lasmiditan failed to decrease carotid artery diameter or blood flow at clinically relevant doses or produce any significant blood pressure changes [43]. 

According to the information included in the Summary Product Characteristics of Reyvow, however, lasmiditan in patients taking also heart rate lowering drugs should be used with caution [28].

### 2.5. Interactions with Central Nervous System (CNS) Depressants

Combined administration of lasmiditan with alcohol and CNS depressants has not been evaluated in clinical trials. Nevertheless, since lasmiditan may result in sedation, other cognitive and/or neuropsychiatric adverse reactions, and driving impairment, it should be used with caution with alcohol and other CNS depressants [14,28,42]. Lasmiditan is highly lipophilic and capable of penetrating the blood–brain barrier (BBB), hence the most common adverse effects of lasmiditan are CNS-mediated effects such as somnolence and fatigue [29,32,36,37,38,39,43]. 

### 2.6. Potential Effect of Lasmiditan on CYP450 Enzymes

Lasmiditan is an in vitro inhibitor of CYP2D6. The in vitro sensitivity analyses suggested that increasing lasmiditan inhibition potential by 10-fold increased desipramine or dextromethorphan (sensitive CYP2D6 substrates) AUC values only by 1.2 to 1.3-fold. The in vitro DDI risk of potential CYP2D6 inhibition (R1) value for CYP2D6 was 1.02, which was right on the cutoff value of 1.02, suggesting that in vivo DDI potential might be low. In other words, the in vivo CYP2D6 inhibition potential for lasmiditan is low [42]. In vitro studies with other CYP enzymes (CYP1A2, CYP2A6, CYP2B6, CYP2C8, CYP2C9, CYP2C19, CYP2E1, or CYP3A4), and non-CYP enzymes (MAO-A, monoamine oxidase A; MAO-B, monoamine oxidase B; FMO3, flavin-containing monooxygenase 3) demonstrated that lasmiditan had a low potential for interactions [42].

It has been shown that lasmiditan does not affect pharmacokinetics of midazolam (CYP3A4 substrate) [41], caffeine (CYP1A2 substrate) [41], or tolbutamide (CYP2C9 substrate) [41], and sumatriptan [40], propranolol [27], or topiramate [39]. 

Summing up, lasmiditan and its metabolites are not clinically relevant inhibitors or inducers for any of the major CYP enzymes [27,39,40,41,42].

### 2.7. Effect of Other Drugs on Lasmiditan’s Pharmacokinetics

Lasmiditan is a substrate for P-gp in vitro; therefore, combined administration of lasmiditan with P-gp inhibitors may result in a potential increase in its blood concentration [14,21,28,35,42]. Lasmiditan, however, is a Biopharmaceutics Classification System (BCS) Class I drug and is unlikely to be affected by P-gp inhibitors [42].

Since lasmiditan undergoes extensive hepatic and extrahepatic metabolism in humans primarily through non-CYP-mediated ketone reduction, inducers and inhibitors of CYP enzymes are unlikely to affect lasmiditan pharmacokinetics [21,29,30,35]. Possible DDIs of lasmiditan are presented in Table S1.

## 3. CGRP Receptor Antagonists—Ubrogepant and Rimegepant 

It has been shown that CGRP—one of the strongest peptides with vasodilating effect, located mainly in the ganglion, nerve and nucleus of the trigeminal nerve—as well as serotonin and dopamine, plays a significant role in a migraine attack [15,16,48,49,50,51]. CGRP and neurotransmitter extravasation occur as a result of dilation of cerebral vessels and functional stimulation within the trigeminovascular system which causes the so-called neurogenic inflammation. It has also been shown that during a migraine attack, along with increasing pain, there is a simultaneous increase in CGRP concentration in the jugular vein [51]. Hence, new groups of drugs directed against CGRP (antagonists of this peptide or its receptor) are a great advancement in migraine therapy [3,15]. 

The beneficial effect of gepants in a migraine attack consists of preventing relaxation of intracranial vessels and not constricting them; therefore, these drugs, as ditans, may be potentially safer than triptans in patients with an increased risk of cardiovascular complications [15,48].

The first gepant for acute treatment of migraine with or without aura in adults, approved by FDA in December 2019, was ubrogepant (UBRELVY, 50 mg and 100 mg tablets, Allergan, Inc., Dublin, Ireland) [52,53].

### 3.1. Ubrogepant 

**Figure 2:**
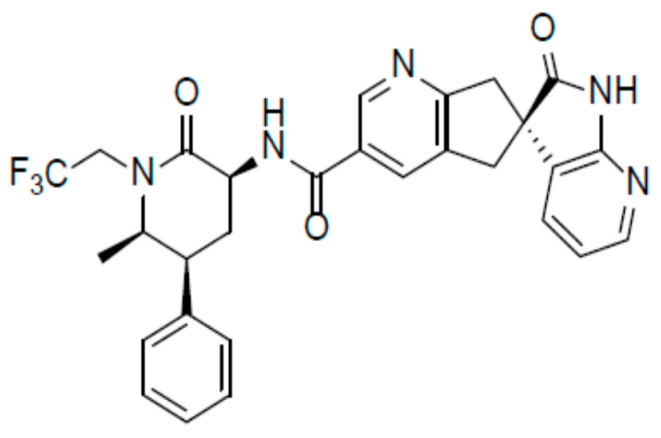
Chemical structure of ubrogepant [53].

The chemical name of ubrogepant is (3′S)-*N*-((3S,5S,6R)-6-methyl-2-oxo-5-phenyl-1-(2,2,2-trifluoroethyl)piperidin-3-yl)-2′-oxo-1′,2′,5,7-tetrahydrospiro[cyclopenta[*b*]pyridine-6,3′-pyrrolo [2,3-*b*]pyridine]-3-carboxamide [52] and has the following structural formula (Figure 2):

The recommended single oral dose of ubrogepant is 50 mg or 100 mg depending on the intensity of pain, up to two doses per day, at least 2 h apart, and the maximum daily dose is 200 mg [52,53,54,55]. Clinical trials [54,55,56,57,58,59,60,61,62,63,64,65] confirmed its efficacy and good tolerance compared to placebo. Adverse reactions reported include mostly rare nausea, insomnia, and dry mouth, and potential hepatotoxicity should be considered [54,55,56,57,58,59,60,61,63,64,65]. 

Upon oral administration, ubrogepant is rapidly absorbed, and C_max_ in plasma is achieved after about 1.5 h. There are no clinically relevant effects of food on pharmacokinetics; as is the case with lasmiditan, however, taking it with food rich in fat may extend the absorption process and T_max_ (up to two hours). The degree of binding of ubrogepant to blood proteins is 87%, biological half-life is 5–7 h, and the drug is mainly excreted in the feces with renal elimination being a minor route (42% and 6% of a radiolabeled dose recovered as parent compound in the feces and urine, respectively). In the case of a single oral dose, the mean apparent central volume of distribution of ubrogepant is about 350 L [52,53,57,63,66,67]. 

Pharmacokinetics of ubrogepant is dose-proportional in the dose range from 1 mg to 400 mg; no accumulation was observed after multiple once daily dosing, and steady state is achieved within 2 days [57,68]. Age, sex, race, bodyweight, as well mild to moderate renal and hepatic impairment have been shown to have no effect on pharmacokinetics (AUC and C_max_) of ubrogepant [57,58,59,64].

#### 3.1.1. Drug–Drug Interactions 

In vitro studies have shown that ubrogepant is neither an inhibitor nor an inducer of the CYP1A2, CYP2B6, and CYP3A4 isoenzymes [53,63]. Following incubations of ubrogepant (0.1–20 µM) with cryopreserved human hepatocytes, it was found that ubrogepant is not an inducer of these three isoenzymes in human incubations at clinically relevant concentrations [63]. It is a weak inhibitor of CYP2C8, CYP2C9, CYP2D6, CYP2C19, MAO-A, and UGT1A1 [57,59,63,65]. Its inhibitory potential in vitro, however, does not appear to be of clinical significance (ubrogepant is not anticipated to be a perpetrator of drug interactions through CYP450s, MAO-A, or UGTA1 inhibition and is not a time-dependent inhibitor of CYP3A4) [63]. The results of in vitro studies also indicate that ubrogepant is a weak substrate of OATP1B1 and OATP1B3 transporters (transfected cells had only 2-fold higher uptake when compared to mock cells). This suggests that significant clinical drug interaction with OATPB1/B3 inhibitors is unlikely [59,63]. 

It is also a weak substrate of OAT1, but not a substrate of OAT3 [57,63]. 

Furthermore, it is not an inhibitor of P-gp, BCRP, BSEP (bile salt export pump), MRP3 (multidrug resistance-associated protein 3), MRP4 (multidrug resistance-associated protein 4), OAT1, OAT3, or NTCP (sodium/taurocholate cotransporting polypeptide) transporters, but a weak inhibitor OATP1B1, OATP1B3, and OCT2 transporters [53,54,57,63]. 

Ubrogepant is a substrate of BCRP and P-gp transporters; therefore, the use of inhibitors of BCRP and/or P-gp may increase exposure of this gepant which is explained in Section BCRP- and/or P-gp-Only Inhibitors below [53,54,55,57,58,63].

##### CYP3A4 Inhibitors 

In vivo studies indicate that ubrogepant should not be used with potent CYP3A4 inhibitors of (e.g., ketoconazole, itraconazole, clarithromycin), as these drugs may cause a significant increase in plasma concentration of ubrogepant which is mainly metabolized by this isoenzyme [53,57,63,66,67]. 

Ketoconazole, for example, caused a 5.3- and 9.7-fold increase in ubrogepant’s C_max_ and AUC, respectively [63].

When ubrogepant is coadministered with moderate (e.g., ciprofloxacin, fluconazole, fluvoxamine, verapamil) inhibitors of CYP3A4, an increase in plasma concentrations of ubrogepant may be observed; hence, its doses should be adjusted. In vivo studies have been demonstrated that C_max_ and AUC of ubrogepant administered with verapamil increased 2.8- and 3.5-fold, respectively [53,63]. 

No interaction studies have been performed to evaluate concomitant use of ubrogepant with weak inhibitors of CY3A4 (ubrogepant can be considered a sensitive CYP3A4 substrate and its exposure is not expected to more than double when used with weak CYP3A4 inhibitors) [57,63]. Based on this, the manufacturer of UBRELVY recommends a starting dose of 50 mg of ubrogepant used concomitantly with moderate or weak inhibitors of CYP3A4 [52]. When used with moderate CYP3A4 inhibitors, a second dose of ubrogepant within 24 h of the starting dose should be avoided. When ubrogepant is used with weak inhibitors of CYP3A4, the second dose of 50 mg may be administered at least 2 h after the first dose, if needed [52,53,63].

Two glucuronide conjugate metabolites of ubrogepant are about 6000-fold less potent in the CGRP receptor binding assay and, thus, are not expected to contribute to pharmacological activity of ubrogepant [53,63]. These metabolites are more hydrophilic than ubrogepant and have only about 30% the exposure of ubrogepant. Therefore, the DDI liability of these metabolites is considered low [63].

##### CYP3A4 Inducers 

Concomitant use of ubrogepant with strong CYP3A4 inducers (e.g., phenytoin, barbiturates, rifampin, St. John’s wort) should be avoided due to ubrogepant’s decreased efficacy [53,57,59,63]. When ubrogepant was administered with rifampin in in vivo studies, its AUC decreased by 80% [63]. Coadministration of ubrogepant with moderate or weak CYP3A4 inducers has not been evaluated in a clinical study. Since ubrogepant is considered a sensitive CYP3A4 substrate, drug interactions in weak or moderate inducers are expected to reduce ubrogepant exposure by 20% to <50% or 50% to <80%, respectively [47,63]. The manufacturer recommends an initial ubrogepant dose of 100 mg when coadministered with moderate or weak CYP3A4 inducers. If needed, a second 100 mg dose of ubrogepant may be administered at least 2 h after the initial dose [52]. Ubrogepant dose adjustment is recommended, however, based on a conservative prediction of 50% reduction in its exposure [63].

##### BCRP- and/or P-gp-Only Inhibitors 

In the case of the combined use of ubrogepant and drugs that inhibit only BCRP and/or P-gp transporters (e.g., quinidine, carvedilol, eltrombopag, curcumin), it is recommended to adjust the dose of ubrogepant [53,57,63,65]. Ubrogepant is a P-gp substrate, and ubrogepant administration with P-gp inhibitors can increase the exposure of ubrogepant. However, no clinical drug interaction studies with inhibitors of these transporters have been performed [53,63]. Drug interaction with verapamil (a combined P-gp inhibitor and moderate CYP3A4 inhibitor) resulted in a 3.5-fold increase in exposure of ubrogepant [63]. This increase can be due to the combined P-gp/CYP3A4 inhibition. Since ubrogepant can be considered a sensitive CYP3A4 substrate, drug interaction due to CYP3A4-only inhibition with moderate CYP3A4 inhibitors such as verapamil is expected to result in at least 2-fold increase in exposure (the range for moderate CYP3A4 inhibitors is 2–5-fold increase in exposure for sensitive CYP3A4 substrates). Therefore, the expected maximum increase in exposure due to P-gp-only inhibition to result in the observed drug interaction will be less than 2-fold. Moreover, the fraction of ubrogepant absorbed is at least 58%. This suggests that the intestinal P-gp inhibition can enhance systemic availability of ubrogepant by about 42%. Therefore, a P-gp-only inhibition is unlikely to result in more than 2-fold increase in ubrogepant exposure [63]. Thus, the manufacturer recommends an initial ubrogepant dose of 50 mg, and, if needed, the second 50 mg dose may be administered at least 2 h after the initial dose [52,53]. An efflux transporter BCRP is expressed in the same tissues as is the case with P-gp. Based on this, the same considerations and dose adjustement recommendations should apply [63]. 

#### 3.1.2. Moderate Food Interaction—Grapefruit Juice 

Due to the fact that grapefruit juice is a moderate inhibitor of CYP3A4, it is expected that it may increase plasma concentration of ubrogepant by inhibiting its metabolism in the intestinal wall and in the liver [52,63,66,67]. The effect of grapefruit juice on ubrogepant metabolism, however, depends significantly on the type of juice and its concentration and may vary. According to the Summary of Product Characteristics of UBRELVY, when coadministered with grapefruit or grapefruit juice, the starting dose of ubrogepant should be 50 mg, and the next dose, if required, should be given 24 h later [52]. 

#### 3.1.3. Other 

There was no evidence of clinically relevant pharmacokinetic interactions of ubrogepant (as a victim or as a perpetrator) when coadministered with the following drugs (as victims or as perpetrators): oral contraceptives (containing norgestimate and ethinylestradiol) [63], acetaminophen [62], naproxen [62], sumatriptan [60], or esomeprazole [63]. Possible DDIs of ubrogepant are summarized in Table S2.

#### 3.1.4. Disease Interactions

Pharmacokinetics of ubrogepant in patients with severe renal impairment (eGFR 15–29 mL/min) or in patients with end-stage renal disease (CLcr < 15 mL/min) have not been studied [53,59,63]. 

In patients with severe renal impairment, doses should be adjusted based on absorption, distribution, metabolism, and elimination information. It must also be assumed that severe renal impairment is unlikely to result in more than a twofold increase in exposure to ubrogepant [63]. No dosing recommendations can be made for patients with end-stage renal disease. 

In patients with severe hepatic impairment (Child–Pugh Class C), ubrogepant exposure was increased by 115%; therefore, dose adjustments in such patients should be made [53,57,59,63]. It was shown that in patients with moderate hepatic insufficiency, C_max_ and AUC of ubrogepant increased by 25% and 52%, respectively, compared to healthy patients [53,57,63].

### 3.2. Rimegepant 

**Figure 3:**
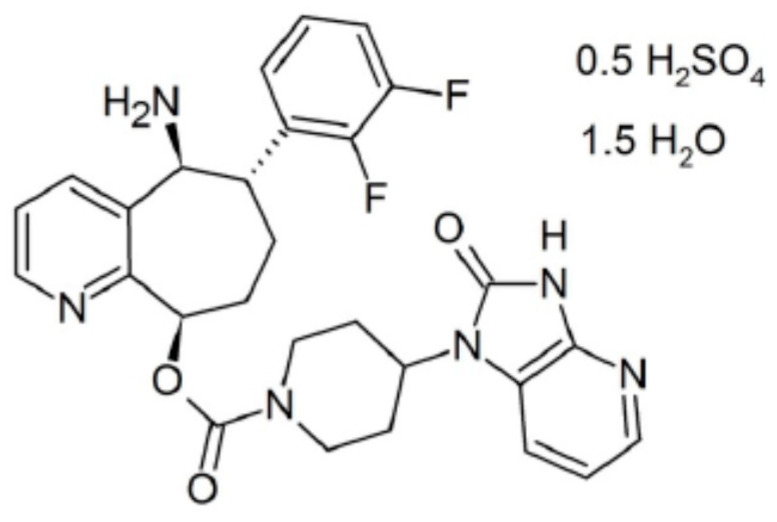
Chemical structure of rimegepant [69].

The second FDA-registered gepant (in February 2020) for use in adult patients in acute treatment of migraine with or without aura was rimegepant (NURTEC ODT, 75 mg the orally disintegrating tablet) [69,70]. Rimegepant sulfate is described chemically as (5S,6S,9R)-5-amino-6-(2,3-difluorophenyl)-6,7,8,9-tetrahydro-5H-cyclohepta[b]pyridine-9-yl 4-(2-oxo-2,3H-imidazo[4,5-b]pyridin-1-yl)piperidine-1-carboxylate [70]. Its chemical structure is presented in Figure 3. NURTEC ODT should be taken on or under the tongue; the recommended dose is 75 mg taken as needed, with a maximum dose of 75 mg over a 24 h period [69,70]. Currently, it is the first and only CGRP receptor antagonist available in the form of orally disintegrating tablets intended for acute treatment of migraine and the only oral CGRP receptor antagonist with effect lasting up to 48 h after a single dose [70].

The most common side effects of rimegepant in clinical trials were mild nausea and urinary tract infection [71,72]. Hypersensitivity reactions, however, including shortness of breath and rash, can occur up to several days after administration. In such an event, rimegepant should be discontinued. In addition, no serious adverse events, including hepatotoxicity, were reported [71,72,73,74,75,76,77,78,79,80,81].

Maximum plasma concentration of rimegepant after oral administration is achieved after 1.5 h, and its bioavailability is 64%. When administered with a high-fat meal, T_max_ of rimegepant was prolonged by 1 h, and C_max_ and AUC were reduced by 42%–53% and by 32%–38%, respectively. The steady-state volume of distribution of rimegepant is 120 L, and plasma protein binding is approximately 96% [69,70,71,72,73,74,75]. Metabolism of rimegepant is mainly mediated by CYP3A4 isoenzyme and to a lesser extent by CYP2C9, resulting in the formation of several minor, inactive metabolites (i.e., metabolites that represented >10% of drug-related material) detected in plasma. Hydroxylation, forming mono- and bis-hydroxylated metabolites, is the most significant biotransformation pathway of rimegepant. Other metabolites excreted are glucuronides, a desaturation product, and an N-dealkylation product [69,70,71,72,73,74,75,80]. About 77% of it is excreted primarily unchanged. Biological half-life is approximately 11 h [69,70,80]. Pharmacokinetics of rimegepant is not affected by age, sex, race/ethnicity, body weight, or CYP2C9 genotype [78].

#### 3.2.1. Drug–Drug Interactions

In vitro studies show that rimegepant is a substrate of CYP3A4 and CYP2C9 [71,72,73,80]. Clinical drug interaction study with midazolam, a sensitive CYP3A4 substrate, indicates that rimegepant is also a weak inhibitor of CYP3A4 with time dependent inhibition. In vitro studies using human liver microsomes indicate that it is not an inhibitor of CYP1A2 (IC50 > 40 µM), 2B6 (IC50 > 40 µM), 2C9 (IC50 > 40 µM), 2C19 (IC50 > 40 µM), 2D6 (IC50 > 40 µM) or UGT1A1 (IC50 > 50 µM), though, or an inducer of CYP1A2, CYP2B6 or CYP3A4 at clinically relevant concentrations [80].

##### CYP3A4 Inhibitors

In in vivo studies, coadministration of a single 75 mg dose of rimegepant with itraconazole, a strong inhibitor of CYP3A4, resulted in a significant increase in rimegepant exposure (AUC increased four-fold and C_max_ approximately 1.5-fold) [80]. Thus, rimegepant can be defined as a moderate sensitive substrate for CYP3A4 (with ≥2 to <5-fold increase in AUC expected with a strong inhibitor of CYP3A4) [47,80]. Therefore, concomitant administration of rimegepant with strong inhibitors of CYP3A4 should be avoided [71,72,73,80]. Since concomitant administration of any moderately sensitive substrate for CYP3A4 with a moderate inhibitor of CYP3A4 is expected to result in exposures increased up to 2-fold [47], a similar increase is also expected with concomitant administration of rimegepant with a moderate inhibitor of CYP3A4 [80]. In a drug interaction study with fluconazole, however, AUC of rimegepant increased about 1.8-fold without significant impact on its C_max_ [80]. Since rimegepant is a substrate of both CYP3A4 and CYP2C9 enzymes, the increased exposure of rimegepant observed can be due to the combined inhibition of CYP2C9 and CYP3A4 with fluconazole. As rimegepant metabolism is primarily mediated by CYP3A4 with lesser contribution from CYP2C9, it is assumed that coadministration of rimegepant with a moderate CYP3A4 inhibitor may increase the AUC of rimegepant less than 2-fold with no significant change in its C_max_.

When rimegepant is concomitantly administered with moderate inhibitors of CYP3A4A, however, administration of the next dose of rimegepant within the next 48 h should be avoided. Coadministration of rimegepant with a weak CYP3A4 inhibitor is not expected to have any clinically significant effect on its exposure [71,72,73,76,77,80].

##### CYP3A4 Inducers

Concomitant use of rimegepant (75 mg single dose) with rifampin, a strong CYP3A4 inducer, reduced its bioavailability (AUC by 80% and C_max_ by 64%), and, thus, also its efficacy [80]. Interaction studies of rimegepant with moderate and weak inducers of CYP3A4 have not been performed, but it is assumed that moderate inducers may also significantly reduce rimegepant exposure by ≥50% to <80%, because rimegepant is a sensitive substrate of CYP3A4 (it will probably be about 50% as rimegepant is a moderately sensitive substrate) [47,80]. Therefore, concomitant administration of rimegepant with strong or moderate inducers of CYP3A is avoided [71,72,73,80].

Drugs that are weak CYP3A4 inducers should not affect bioavailability of rimegepant. Weak inducers of CYP3A4 may decrease exposures of a sensitive substrate of CYP3A4 by ≥20% to <50% [47]. Since rimegepant is a moderately sensitive substrate of CYP3A4, its exposures may decrease by about 20% after concomitant administration of any weak inducer of CYP3A4 [80].

##### CYP2C9 Inhibitors

As described above, administration of rimegepant as a single 75 mg dose with fluconazole (a moderate inhibitor of both CYP3A4 and CYP2C9) increased rimegepant exposure (AUC 1.8-fold) with no significant effect on C_max_ [80]. Rimegepant is metabolized mainly by CYP3A4 and, to a lesser extent, by CYP2C9 [69,71,72,73,80]. The increase in rimegepant exposure, in this case, was attributable to concomitant inhibition of CYP2C9 and CYP3A4, suggesting a small contribution of CYP2C9. In addition, since rimegepant is primarily eliminated in an unchanged form with no active metabolites [71,72,73,80] it is less likely that the concomitant administration of rimegepant with inhibitors of CYP2C9 would result in considerable increase in its exposure. Therefore, inhibition of CYP2C9 only is not expected to have a significant effect on exposure of rimegepant [80].

##### Membrane Transporters

Since rimegepant is a substrate of P-gp and BCRP efflux transporters, coadministration of this drug with inhibitors of P-gp or BCRP may significantly increase its exposure [69,70,71,72,73,78,80]. In in vitro studies, the secretory transport of rimegepant was inhibited by co-incubation with the inhibitors of P-gp (ketoconazole and cyclosporine A) indicating that concomitant administration of rimegepant with inhibitors of P-gp may increase the exposure of rimegepant [80]. Rimegepant was found to be a BCRP substrate in the bi-directional transport assays conducted using MDCKII (Madin-Darby Canine Kidney) cells expressing the human BCRP. The efflux ratio was >2.0 and was reduced by >50% in the presence of BCRP transport inhibitors [80]. No clinical studies were conducted. Therefore, rimegepant should not be used with inhibitors of P-gp (e.g., quinidine) or BCRP (e.g., eltrombopaq, curcumin) [69,73,80].

Rimegepant is not a substrate of OATP1B1 or OATP1B3, nor is it an inhibitor of P-gp [69,73,80] (half maximum inhibitory concentration (IC50) is > 100 µM), BCRP (IC50 > 50 µM), OAT1 (IC50 > 10 µM) or MATE2-K (IC50 > 10 µM). It is an inhibitor of OATP1B3 (IC50 is 6.04 µM), OCT2 (IC50 is 1.08 µM) and MATE1 (IC50 is 1.18 µM) and a weak inhibitor of OATP1B1 (11% at 5 µM), and OAT3 (24% at 5 µM) [80]. Based on in vitro studies, clinically relevant drug interactions of rimegepant are less likely. For OAT, OCT and MATE the ratio of maximal unbound plasma concentration of the interacting drug at steady state to maximal inhibitory concentration (Imax,u/IC50) is ≤0.06 [80]. Clinical studies, also, did not indicate elevated serum creatinine levels over time in participants receiving therapeutic doses of rimegepant suggesting interaction with OCT2, OAT2, and MATEs [73,80].

##### Other—Rimegepant as a Perpetrator

In vitro studies indicated that rimegepant was a weak to moderate time-dependent inhibitor of human CYP3A4 [73,80]. In drug interaction studies, no pharmacokinetic interactions were observed when rimegepant was coadministered with oral contraceptives (norelgestromin, ethinylestradiol) [80] and midazolam [80]. Increase in the exposure of midazolam at steady-state was below 2-fold, indicating that rimegepant could be classified as a weak inhibitor of CYP3A4 [80]. Concomitant administration of rimegepant (at steady-state) and sumatriptan (single-dose) did not affect pharmacokinetics of any of these drugs [80,82]. Moreover, no considerable differences were observed in the time-weighted average of mean arterial pressure between sumatriptan alone and sumatriptan coadministered with rimegepant [82]. Possible DDIs of rimegepant are shown in Table S3.

#### 3.2.2. Disease Interactions

Use of rimegepant should be avoided in patients with severe hepatic impairment who may have significantly higher levels of rimegepant (up to twofold) [69,73,80]. No dose adjustment is required for patients with mild (Child–Pugh A) or moderate (Child–Pugh B) hepatic impairment [69]. Rimegepant has not been administered to patients with end-stage renal disease (CLcr < 15 mL/min) or to patients on dialysis; therefore, its use should be avoided in these patients. No dosage adjustment of rimegepant is required for patients with mild, moderate, or severe renal impairment [69,76,77,79,80].

#### 3.2.3. Moderate Food Interaction—Grapefruit Juice

Similar to ubrogepant, concomitant administration of rimegepant with grapefruit or grapefruit juice—CYP3A4 inhibitors—may increase its plasma levels. The effect of grapefruit juice is concentration, dose, and formulation dependent and can vary greatly between brands [74,75].

Therefore, patients receiving rimegepant should rather avoid regular intake of grapefruits and grapefruit juice to prevent an excessive increase in plasma levels of this drug. When consuming grapefruit or grapefruit juice with rimegepant, it is recommended that the next dose of rimegepant is not administered earlier than 48 h afterwards [74,75,80].

## 4. Anti-CGRP Monoclonal Antibodies (mAbs)

Four mAbs directed against the CGRP receptor (erenumab) [83] and against the CGRP peptide itself (fremanezumab [84], galcanezumab [85], eptinezumab [86]) are now approved for preventive treatment of chronic migraine (headaches for at least four days per month and intolerance or insufficient response to medications used prophylactically (e.g., topiramate, beta-blockers, anticonvulsants or antidepressants). As shown in clinical trials [87,88,89,90,91,92,93,94,95,96,97,98,99,100,101,102,103,104,105,106,107,108,109], all of the above mAbs cause an over 50% reduction in the number of pain days in patients with chronic migraine, including patients with a headache caused by overuse of analgesics. Clinical studies also confirm their favorable safety profile, and the most common side effects are skin reactions (rash, itching) at the injection site. One should bear in mind, however, that angioedema and anaphylactic reactions have also been reported. Hypersensitivity reactions can occur within minutes of drug administration but also more than one week after treatment. If a hypersensitivity reaction occurs, consideration should be given to discontinuing the use of the monoclonal antibody [87,88,89,90,91,92,93,94,95,96,97,98,99,100,101,102,103,104,105,106,107,108,109,110]. Mild or moderate constipation may also be a common side effect of erenumab. Most constipation cases begin after the first dose of treatment but can also arise later and usually resolve within three months of starting erenumab treatment [88,89]. Anti-CGRP monoclonal antibodies are less hepatotoxic than gepants, their metabolism is based on reticuloendothelial uptake. Since monoclonal antibodies are not known to be eliminated via renal pathways or metabolized in the liver, renal and hepatic impairment are not expected to impact their pharmacokinetics [111,112,113,114,115,116,117,118].

### 4.1. Pharmacokinetics and Pharmacodynamics of mAbs and the Risk of Interactions

Bioavailability and absorption rate of mAbs depend, among other things, on the route of administration. Due to their protein structure, mAbs are transported in the body by endocytosis, pinocytosis, or passive transport through the pores in intercellular space [111,118]. The lymphatic system plays an active part in mAb absorption and the main route of transport after subcutaneous (s.c.) administration is lymph, unlike small molecule drugs that are transported through blood plasma. Therefore, one of the key factors affecting bioavailability of mAbs and their absorption rate upon s.c. administration is the transit time of mAbs with the lymph [111,112,113,114,118].

Prolonged absorption of mAbs from the injection site after s.c. administration is the cause of the flip-flop effect, and the peak concentration usually takes about 5–8 days of drug administration [90,97,98,101,108,111].

mAbs are distributed mainly by lymph and blood, while redistribution with bile or saliva is not observed. mAbs penetrate poorly into the tissues, and after passing through the vascular endothelium they additionally bind with components of the intracellular fluid which hinders further distribution [111]. Unlike small-molecule drugs, the pharmacodynamic phase of mAbs significantly affects their pharmacokinetics. For small-molecule drugs, the processes related to interaction with the receptor are of little importance. In contrast, for mAbs, binding to receptors for both the Fc and Fab fragments of the antibody significantly modifies the drug’s distribution. The arm exchange process plays a unique role in distribution of mAbs based on the IgG4 structure, e.g., galcanezumab (after administration to the blood, mAbs of the IgG4 subclass are exchanged in one of the arms with natural antibodies of the IgG4 subclass) [111,118]. One of the critical mechanisms involved in mAbs distribution is the reversible binding to the neonatal Fc receptor (FcRn) to which all IgG antibodies bind. This bond is not permanent, and the mechanism itself depends, among others, on pH, while the formation of the mAb–FcRn complex is seen as the leading cause of the long half-life of mAbs [111]. This is because FcRn, which is constitutively expressed in the vascular endothelium, binds to IgG in a pH-dependent manner protecting it from lysosomal degradation, and then recycles IgG by receptor-mediated endocytosis. The mechanism is as follows: IgG is taken up into cells by non-specific fluid-phase pinocytosis (the intestinal surrounding fluid is captured on the apical surface of the enterocyte) and trafficked to the early endosome. As the endosome acidifies (pH 6.0), IgG binds to FcRn. Unbound IgG undergoes degradation in the lysosome, while IgG–FcRn complexes are recycled back to the cell surface. At a physiological pH, IgG–FcRn complex dissociates and releases IgG back to circulation, which protects IgG from lysosomal degradation, thus increasing its half-life. This phenomenon has been leveraged to increase the half-life of mAbs by optimizing the strength at which IgG binds to FcRn in the acidic endosomal environment. Importantly, the above mechanism is not easily saturated at therapeutic mAbs concentrations [118].

The volume of distribution for antibodies usually does not exceed the volume of the central compartment (3–7 L) [90,97,98,101,108,111].

An essential element affecting metabolism of mAbs is their binding via the Fc fragment of the mAb to receptors present on leukocytes and other cells involved in immune responses. Due to mAbs binding via the Fc fragment, the humoral and cellular responses after mAbs administration are modified. The key receptors interacting with mAbs include the FcRn receptor, the mannose receptor (MR), the asialoglycoprotein receptor (ASGPR), and the Fcγ class of receptors (FcγR) [111,118,119,120].

Significant modifications to the structures significantly affecting pharmacokinetics of mAbs include fucosylation and galactosylation. Low fucosylation and galactose exposure are associated with increased antibody-dependent cellular cytotoxicity (ADCC) [111]. This is due to the greater affinity of nonfucosylated mAbs for FcγRIIIa. Kidneys and the liver are not involved in the elimination and metabolism of mAbs. Any damage to the liver or kidneys, however, may result in activation of innate and adaptive immune responses through secretion of pro-inflammatory cytokines and chemokines [111,118].

Generally, mAbs administered with concomitant medications are not expected to result in clinically relevant pharmacokinetic interactions and are unlikely to affect drug-metabolizing enzymes or transporters because they are metabolized by general proteolytic degradation pathways [90,97,98,101,108,111].

Interactions between small molecule drugs and mAbs are thus observed in the pharmacodynamic phase associated with modulation of the immune system function, rather than in the pharmacokinetic phase. Modulation of the immune system changes characteristics of the receptor clearance, which may lead to clinically significant changes in kinetics of mAbs, e.g., a decreased expression of the target antigen, a decreased expression of the FcγRI receptor, an increased ability to saturate the FcRn receptor, and reduced ADA generation (e.g., the interaction between adalimumab and methotrexate) [111,119]. In the case of small molecule drugs, their metabolism may be modified due to the effect of mAbs on microsomal cytochrome P450 enzymes (e.g., the interaction between adalimumab and duloxetine) [111].

Pharmacodynamic interactions are related to the effects of immunomodulatory drugs on expression of Fcγ receptors. Such an interaction takes place in the case of the use of itracoazole, for instance, which affects expression of Fc receptors, where the group of FcγR is one of the central elements of communication between mAbs and the immune system. Depending on the class of IgG used to produce the mAbs, modulation of FcγR expression on immunocompetent cells may have a significant effect on pharmacokinetics/pharmacodynamics of mAbs used concurrently [111,118].

Repeated administration of mAbs is known for its potential to be highly immunogenic [119,120]. The immunogenicity of mAbs is manifested in production of anti-drug antibodies (ADAs), in some cases in as much as 70% of patients [119]. Antibodies to mAbs generally appear within the first 28 weeks of treatment. Even the use of complete human antibody genes has not completely eliminated immunogenicity and ADAs’ associated induction [119]. ADAs can alter pharmacokinetics (mainly by affecting elimination and extending or reducing biological half-life) and pharmacodynamics of mAbs, reducing their efficacy or even completely neutralizing their therapeutic effects and causing the patient to experience serious adverse events [118,119,120].

Production of ADAs depends on many patient-related factors (genetic background, co-treatment, disease state) and the drug (dose, frequency, route of administration, impurities, formulation, post-translational modifications, antibody origin, mAb target) [119]. Multiple injections and higher doses of mAbs generally increase the risk of ADAs, although not universally [119,121,122]. Immunogenicity profiles of erenumab [88], fremanezumab [123], and galcanezumab [124] show no effect of ADA formation on mAbs’ efficacy or safety, or on pharmacokinetics of galcanezumab [113]. Reported immunogenicity rates for these three mAbs were relatively low at 8.9% [90,119,125,126], 0.4–1.6%, and 12.5% for erenumab, fremanezumab, and galcanezumab, respectively [119].

### 4.2. Erenumab

Erenumab (AIMOVIG, solution for subcutaneous injection in a pre-filled syringe or pre-filled pen, 70 mg and 140 mg, Novartis Europharm Limited) was the first monoclonal antibody for treatment of chronic migraine in adults, approved by the FDA and the EMA. It is a fully human IgG2 monoclonal antibody produced using recombinant DNA technology in Chinese hamster ovary (CHO) cells [83]. The recommended dose is 70 mg or 140 mg s.c. once a month, and clinical improvement usually is achieved within three months [83,88,89]. Erenumab’s bioavailability upon s.c. administration is 82%, maximum plasma concentrations are reached after 4–6 days (from 3 to 14 days) [16,83,88,89], steady-state is achieved after 12 weeks, and biological half-life is 28 days [88,89]. Erenumab is eliminated in two phases: at low concentrations, elimination mainly takes place by saturable binding to the target CGRP receptor, and at higher concentrations, mainly by non-specific proteolysis. Throughout administration, erenumab is eliminated primarily by non-specific proteolysis [88,89,90].

No interaction with oral contraceptives (ethinyl estradiol/norgestimate) or sumatriptan was observed in studies with healthy volunteers. Erenumab has no relevant food–drug interactions [83,88,89,90].

### 4.3. Fremanezumab

The second monoclonal antibody to be approved by the FDA and EMA for migraine prevention was fremanezumab (AJOVY, solution for subcutaneous injection, 225 mg in pre-filled syringes) [84]. Fremanezumab is a humanised IgG2Δa/kappa monoclonal antibody derived from a murine precursor. It can be used according to two dosing schedules: 225 mg once a month or 675 mg every three months (three injections of 225 mg). When changing the dosing schedule, the first dose of the new schedule should be administered at the next scheduled dosing date. Clinical efficacy should be evaluated after three months. Maximum plasma concentration of fremanezumab after a single administration is reached after 5–7 days (from 3 to 20 days) [16,84,91,92,93,94,95,96,97], and the absolute bioavailability is 55% and 66% for 225 mg and 900 mg, respectively. A steady-state is achieved within approximately 168 days [91,92,93,94,95,96,97]. There is no need to modify the dosage in the elderly or in patients with mild to moderate renal or hepatic impairment [92,93]. Using population pharmacokinetic modeling and simulation of fremanezumab in healthy subjects and patients with migraine, it was shown, however, that higher body weight was associated with a lower exposure of fremanezumab (an increased central clearance and distribution volume) [96].

Concomitant use of acute migraine treatments (analgesics, ergots, and triptans) and migraine preventive medicinal products during the clinical studies did not affect pharmacokinetics of fremanezumab [92,93].

### 4.4. Galcanezumab

Galcanezumab (EMGALITY, solution for subcutaneous injection, 120 mg in pre-filled pen) is a recombinant humanised monoclonal antibody produced in CHO cells. The FDA and EMA approved the drug; the recommended dosage is one subcutaneous injection at a dose of 120 mg once a month with the first loading dose of 240 mg [113,114,115]. Galcanezumab has been shown to be effective also in prevention of cluster headache attacks [99,105].

The time to reach maximum serum concentration of galcanezumab is 5 days (from 7 to 14 days), the apparent volume of distribution is 7.3 L, and the half-life is 27 days [98,100,101]. Galcanezumab exposure increases proportionally with dose. The population pharmacokinetic analysis, which included galcanezumab doses from 5 mg to 300 mg, absorption rate, apparent clearance, and apparent volume of distribution, was independent of the dose. No pharmacokinetic drug interactions are expected [98,100,101,102,103,104,106,107].

### 4.5. Eptinezumab

Eptinezumab (VYEPTI, 100 mg ampoules) was approved by the FDA in February 2020 and is the first drug in its class to be administered intravenously for migraine attacks [116]. The recommended dosage is 100 mg in an i.v. infusion over 30 min or a maximum of 300 mg every three months [108,109,110].

Eptinezumab exhibits linear pharmacokinetics, with exposure increasing proportionally with the dose (from 100 mg to 300 mg) following intravenous administration. Steady-state plasma concentrations are achieved with the first dose of the once every three months dosing schedule. Eptinezumab’s distribution volume is approximately 3.7 L, and biological half-life is about 27 days [117].

Interactions with concomitant drugs that are substrates, inducers, or inhibitors of cytochrome P450 enzymes are unlikely [108,109,110,117].

There is no pharmacokinetic interaction with sumatriptan. Coadministration of a single dose of 300 mg of eptinezumab (intravenous infusion over 1 h ± 15 min) with a single dose of 6 mg of sumatriptan administered subcutaneously did not significantly affect pharmacokinetics of eptinezumab or sumatriptan [117].

## 5. Conclusions

Clinical study results have demonstrated that lasmiditan, gepants, and mAbs approved for migraine treatment in the last two years are highly effective and generally well tolerated. Moreover, mAbs do not show any pharmacokinetic interactions with other drugs. Nevertheless, monitoring efficacy and safety of mAbs, including ADA levels in patient serum, as well as the presence of neutralizing antibodies that interfere with biological and clinical activity of mAbs can help determine the causes of the loss of response and provide the basis for treatment modification. In the case of lasmiditan, special attention should be paid to combining it with serotonergic drugs. Strong CYP3A4 inhibitors and CYP3A4 inducers, meanwhile, should not be used with rimegepant and ubrogepant.

These new drugs give millions of patients suffering from migraine the hope for a better quality of life, reduced frequency of the attacks, and, thus, reducing the number of drugs they take. Thereby, they decrease the risk of developing DDIs and of secondary drug overuse headaches. According to recent reports, the currently recommended anti-migraine therapies, including anti-CGRP mAbs, may also be indicated for treatment of headaches in patients with COVID-19.

## Supplementary Materials

The following are available online at https://www.mdpi.com/1999-4923/12/12/1180/s1, Table S1: Possible drug-drug interactions of lasmiditane, Table S2: Possible drug-drug interactions of ubrogepant, Table S3: Possible drug-drug interactions of rimegepant.
