# Supplementary material for: Pharmacokinetics, Pharmacodynamics and Drug–Drug Interactions of New Anti-Migraine Drugs—Lasmiditan, Gepants, and Calcitonin-Gene-Related Peptide (CGRP) Receptor Monoclonal Antibodies
Source: Pharmaceutics. 2020 Dec 3;12(12):1180. doi: 10.3390/pharmaceutics12121180 (PMC7761673; doi:10.3390/pharmaceutics12121180)
Supplement: Supplementary file 1 [file pharmaceutics-12-01180-s001.pdf]

pharmaceutics

MDPI

Supplementary

Pharmacokinetics, Pharmacodynamics and Drug-Drug Interactions of New Anti-Migraine Drugs–Lasmiditan, Gepants, and Calcitonin-Gene-Related Peptide (CGRP) Receptor Monoclonal Antibodies

Danuta Szkutnik-Fiedler

Table S1. Possible drug-drug interactions of lasmiditan [14,28,31,35–38,40,42,45–47].

The risk or severity of serotonin syndrome can be potentially increased when lasmiditan is combined with the following drugs[1,♣]	Serum concentration of the following drugs (P-gp and/or BCRP substrates) may potentially increase when combined with lasmiditan[2,♣]	Serum concentration of lasmiditan (P-gp substrate) may potentially increase when it is combined with the following drugs[3,♣]	Lasmiditan may increase the bradycardic effects of the following drugs[♣]
5-hydroxytryptophan*	afatinib		acebutolol
alfentanil*	alpelisib		amlodipine
almotriptan*	ambrisentan		atenolol
amitriptiline*	apixaban		betaxolol
amoxapine*	belinostat		carteolol
buspirone*	bisoprolol		carvedilol
citalopram*	brentuximab vedotin		diltiazem
clomipramine*	cabazitaxel		esmolol
cyclobenzaprine*	ceritinib		felodipine
desipramine*	cladribine		isradipine
desvenlavaxine*	cobimetinib	clobazam	ivabradine
dexfenfluramine*	colchicine*	daclatasvir	labetalol
dextromethorphan*	cyclosporine	erythromycin	levobetaxolol
dihydroergotamine*	daunorubicin	fexofenadine	levobunolol
dolasetron*	delafloxacin	lapatinib	methyldopa
doxepin*	digitoxin	ritonavir	metipranolol
doxepin topical*	digoxin		metoprolol
duloxetine*	donepezil		nadolol
eletriptan*	doxorubicin		nebivolol
ergotamine*	edoxaban*		nicardipine
escitalopram*	etoposide		nifedipine
fenfluramine*	everolimus		nimodipine
fentanyl*	idelalisib		nisoldipine
fluoxetine*	imatinib		propranolol
fluvoxamine*	indacaterol		rivastigmine
frovatriptan*#	irinotecan		siponimod
furazolidone*	lemborexant		timolol

granisetron*	linagliptin
imipramine*	lusutrombopag
isocarboxazid*	methotrexate
levomilnacipran*	mibefradil
linezolid*	mirabegron
lorcaserin*	mitoxantrone
maprotiline*	nilotinib
meperidine*	nintedanib
methylene blue*	osimertinib
milnacipran*	ozanimod*
mirtazapine*	posaconazole
naratriptan*#	prucalopride
nefazodone*	quinidine
nortriptyline*	ranolazine
ondansetron*	rifaximin
palonosetron*	rimegepant
paroxetine*	riociguat
pentazocine*	rosuvastatin
phenelzine*	saquinavir
procarbazine*	selexipag
protriptyline*	sofosbuvir
rasagiline*	sulfasalazine
remifentanil*	tenofovir
rizatriptan*#	tenofovir alafenamide
safinamide*	tenofovir disoproxil
selegiline*	teriflunomide
sertraline*	topotecan
sibutramine*	vemurafenib
St. John's wort*	vinblastine
sufentanil*	vincristine
tapentadol*	
tranylcypromine*	
trazodone*	
trimipramine*	
tryptophan*	
venlafaxine*	
vilazodone*	
vortioxetine*	
zolmitriptan*#	

* Major interaction according to the www.drugs.com [46]; ♣ Information about the interaction can be found on both www.drugs.com [46] and www.drugbank.com [45]; [1] Lasmiditan is a direct serotonin receptor agonist. When administered concomitantly with other drugs that also increase 5-HT levels the risk of serotonin syndrome increases [14,28,31,35–38]. No clinical DDI studies were conducted to evaluate the clinical significance of these drug-drug interactions, except sumatriptan; # Coadministration of lasmiditan and sumatriptan did not cause serotonin syndrome or any clinically relevant interaction between these two drugs [40]; [2] Lasmiditan exhibited *in vitro* inhibition of intestinal P-gp and BCRP with drug-drug interaction indices I_{gut}/IC_{50} exceeded the FDA [47] cutoff value of 10, indicating that lasmiditan has the potential to inhibit P-gp or BCRP *in vivo* [42]. No clinical DDI studies were conducted; [3] Lasmiditan is a substrate for P-gp *in vitro*; therefore, combined administration of lasmiditan with P-gp inhibitors may result in a potential increase of its blood

concentration [14,21,28,35,42]. Lasmiditan, however, is a BCS Class I drug and is unlikely to be affected by P-gp inhibitors [42]. No clinical DDI studies were conducted.

Table S2. Possible drug-drug interactions of ubrogepant [45,46,53–55,57–59,63,65–67].

Serum concentration of ubrogepant may potentially increase when it is combined with the following drugs (BCRP and/or P-gp inhibitors)[1,♣]	Serum concentration of ubrogepant may potentially increase when it is combined with the following drugs (CYP3A4 inhibitors)[2,♣]	Serum concentration of ubrogepant may potentially decrease when it is combined with the following drugs (CYP3A4 inducers)[2,♣]
afatinib	abametapir topical	aminogluthethimide
bepridil	abiraterone	amobarbital
capmatinib	amiodarone	apalutamide[*]
carvedilol	amlodipine	armodafinil
curcumin	amprenavir	bexarotene
cyclosporine	aprepitant	bosentan
daclatasvir	atazanavir[*]	brigatinib
eliglustat	bicalutamide	butabarbital
eltrombopaq	boceprevir[*]	butalbital
eluxadoline	ceritinib[*]	carbamazepine[*]
encorafenib	chloramphenicol	cenobamate
flibanserin	cimetidine	dabrafenib
fostamatinib	ciprofloxacin	dexamethasone
fostemsavir	clarithromycin[*]	doravirine
gefitinib	clotrimazole	efavirenz
ibrutinib	cobicistat[*]	elagolix
lapatinib	conovaptan	enzalutamide[*]
lasmiditan	crizotinib	eslicarbazepine
midostaurin	danazol	etravirine
neratinib	darunavir	felbamate
niraparib	delavirdine[*]	fosphenytoin[*]
olaparib	diltiazem	glycerol phenylbutyrate
osimertinib	duvelisib	griseofulvin
ponatinib	erythromycin	ivosidenib
progesterone	fedratinib	lesinurad
propafenone	fluconazole	lorlatinib
quinidine	fluvoxamine	mephobarbital
quinine	fosamprenavir	metreleptin
regorafenib	fosaprepitant	mitotane[*]
rolapitant	goldenseal	modafinil
safinamide	grepafloxacin	nafcillin
sarecycline	idelalisib[*]	nevirapine
simeprevir	imatinib	oritavancin
sonidegib	indinavir[*]	oxcarbazepine
spironolactone	isavuconazonium	pentobarbital
tacrolimus	isoniazid	pexidartinib
tamoxifen	itraconazole[*]	phenobarbital[*]

tedizolid	ivacaftor	phenytoin*
teriflunomide	ketoconazole*	pitolisant
ticagrelor	lefamulin	primidone*
tolvaptan	lemborexant	rifabutin*
ulipristal	letermovir	rifampin*
uridine	levamlodipine	rifapentine*
valbenazine	lomitapide	rufinamide
venetoclax	mibefradil*	secobarbital
	mifepristone*	somapacitan-beco
	nefazodone*	somatrem
	nelfinavir*	somatropin
	nifedipine	St. John's wort*
	nilotinib	tazemetostat
	palbocilib	telotristat
	pazopanib	troglitazone
	posaconazole*	vemurafenib
	ranolazine	
	ribocilib	
	ritonavir*	
	rucaparib	
	saquinavir*	
	selpercatinib	
	stiripentol	
	suvorexant	
	telaprevir*	
	telithromycin*	
	troleandomycin*	
	tucatinib	
	verapamil	
	voriconazole*	
	voxelotor	
	zafirlukast	

* Major interaction according to the www.drugs.com [46] (strong CYP3A4 inhibitors or strong CYP3A4 inducers); * Major interaction according to the www.drugs.com [46] (strong CYP3A4 inhibitors or strong CYP3A4 inducers); ♣ Information about the interaction can be found on both www.drugs.com [46] and www.drugbank.com [45]; [1] No clinical DDI studies with inhibitors of P-gp and BCRP transporters have been performed [53,63]; [2] DDI studies with CYP3A4 modulators were conducted for verapamil, ketoconazole, and rifampin [63].

Recommended dosage:

i. BCRP and/or P-gp inhibitors: an initial ubrogepant dose is 50 mg, a second 50 mg dose may be administered at least 2 hours after the initial dose;
ii. strong CYP3A4 inhibitors: combinations should be avoided;
iii. moderate or weak CYP3A4 inhibitors: an initial ubrogepant dose is 50 mg, a second 50 mg dose should be avoided within 24 hours of the initial dose (moderate CYP3A4 inhibitors), a second 50 mg dose may be administered at least 2 hours after the initial dose (weak CYP3A4 inhibitors);
iv. strong CYP3A4 inducers: combinations should be avoided;

v. moderate or weak CYP3A4 inducers: an initial ubrogepant dose is 100, a second 100 mg dose may be administered at least 2 hours after the initial dose.

Table S3. Possible drug-drug interactions of rimegepant [45,46,69,71–73,76–78,80,82].

Serum concentration of rimegepant may potentially increase when it is combined with the following drugs (BCRP and/or P-gp inhibitors)[1,♣]	Serum concentration of rimegepant may potentially increase when it is combined with the following drugs (CYP3A4 inhibitors)[2,♣]	Serum concentration of rimegepant may potentially decrease when it is combined with the following drugs (CYP3A4 inducers)[2,♣]
afatinib<br>amiodarone<br>capmatinib<br>cobimetinib<br>cyclosporine<br>eltrombopaq<br>encorafenib<br>fostemsavir<br>gefitinib<br>ibrutinib<br>istradefylline<br>lapatinib<br>lasmiditan<br>midostaurin<br>neratinib<br>niraparib<br>olaparib<br>osimertinib<br>ponatinib<br>propafenone<br>regorafenib<br>rolapitant<br>safinamide<br>sarecycline<br>sonidegib<br>sulfasalazine<br>tacrolimus<br>tafamidis<br>tedizolid<br>teriflunomide	abametapir topical<br>abiraterone<br>amprenavir<br>aprepitant<br>atazanavir*<br>boceprevir*<br>ceritinib*<br>chloramphenicol<br>ciprofloxacin<br>clarithromycin*<br>cobicistat*<br>conovaptan<br>crizotinib<br>darunavir<br>delavirdine*<br>diltiazem<br>dronedarone<br>duvelisib<br>erythromycin<br>fedratinib<br>fluconazole<br>fosamprenavir<br>fosaprepitant<br>idelalisib*<br>imatinib<br>indinavir*<br>isavuconazonium<br>itraconazole*<br>ivacaftor<br>ketoconazole*<br>letermovir<br>mibefradil*<br>nefazodone*<br>nelfinavir*<br>palbocilib<br>posaconazole*<br>ranolazine<br>ribocilib<br>ritonavir*	apalutamide*<br>bosentan*<br>carbamazepine*<br>cenobamate*<br>dabrafenib*<br>dexamethasone*<br>efavirenz*<br>enzalutamide*<br>eslicarbazepine*<br>etravirine*<br>fosphenytoin*<br>lorlatinib*<br>mitotane*<br>modafinil*<br>nafcillin*<br>nevirapine*<br>pexidartinib*<br>phenobarbital*<br>phenytoin*<br>primidone*<br>rifabutin*<br>rifampin*<br>rifapentine*<br>somapacitan-beco<br>somatrem<br>St. John's wort*<br>telotristat*<br>vemurafenib

rucaparib
saquinavir*
selpercatinib
stiripentol
telaprevir*
telithromycin*
troleandomycin*
tucatinib
verapamil
voriconazole*
voxelotor

* Major interaction according to the www.drugs.com [46] (strong CYP3A4 inhibitors or strong CYP3A4 inducers); ♣ Information about the interaction can be found on both www.drugs.com [46] and www.drugbank.com [45]; [1] No clinical DDI studies with inhibitors of P-gp and BCRP transporters have been performed [80]; [2] DDI studies with CYP3A4 modulators were conducted for itraconazole and rifampin [80].

Recommended dosage:

i. BCRP and/or P-gp inhibitors: an initial rimegepant dose is 50 mg, a second 50 mg dose may be administered at least 2 hours after the initial dose;
ii. strong CYP3A4 inhibitors: combinations should be avoided;
iii. moderate CYP3A4 inhibitors: an initial rimegepant dose is 50 mg, a second 50 mg dose should be avoided within 24 hours of the initial dose;
iv. weak CYP3A4 inhibitors: an initial rimegepant dose is 50 mg, a second 50 mg dose may be administered at least 2 hours after the initial dose;
v. strong or moderate CYP3A4 inducers: combinations should be avoided;
vi. weak CYP3A4 inducers: an initial rimegepant dose is 100, a second 100 mg dose may be administered at least 2 hours after the initial dose.
